# Iatrogenic Pneumothorax with Subsequent Subcutaneous Emphysema

**DOI:** 10.7759/cureus.6480

**Published:** 2019-12-27

**Authors:** Eric Yuschak, George Michael, Jesus Lanza, Furqan Haq

**Affiliations:** 1 Family Medicine, St. Petersburg General Hospital, St. Petersburg, USA; 2 Internal Medicine, St. Petersburg General Hospital, St. Petersburg, USA; 3 Intensive Care, St. Petersburg General Hospital, St. Petersburg, USA; 4 Internal Medicine, Oak Hill Hospital, Tampa, USA

**Keywords:** hemoptysis, lung cancer, pneumothorax, subcutaneous emphysema

## Abstract

This presentation reports a case of a 67-year-old former smoker who presented to the emergency department with new-onset hemoptysis. During the workup, a left lung mass was identified. During the biopsy, he experienced a pneumothorax. The procedure had to be aborted, and a small-sized chest tube was placed. The following day, the patient underwent a successful second lung biopsy, but a day later he developed significant subcutaneous emphysema despite having a chest tube. The same day, the smaller chest tube was removed and a larger chest tube was inserted. While small chest tubes are preferred for patient comfort, in some patients with risk factors, a large chest tube is recommended. Over the course of a few days, the emphysema improved.

## Introduction

Hemoptysis is defined as coughing up of blood that originates from the airways (the lungs or bronchioles). The most common causes of hemoptysis include infectious, which accounts for 60-70% of cases, followed by lung malignancies, which account for 23% [1]. Infectious causes include acute and chronic bronchitis, pneumonia, and tuberculosis. The condition must also be differentiated from pseudo-hemoptysis, which is coughing up of blood not from the lungs or bronchioles, as well as from hematemesis, which is vomiting containing blood. The workup for hemoptysis starts with a chest radiograph. Additional workup may include bronchoscopy and/or computer tomography (CT) scan if there is suspicion for a mass or bronchial lesion [2]. The goals of the workup and treatment include bleeding cessation, aspiration prevention, airway management, and finding an underlying cause.

Lung cancer is the leading cause of cancer-related death in the United States, accounting for 13% of all new cancer diagnoses and 24% of cancer deaths [3]. The primary risk factor for lung cancer is tobacco use including former use and secondhand exposure, with relative risk decreasing accordingly from 20 to 9 to 1.3 [4]. The primary tumor typically presents with chest discomfort, cough, dyspnea, and hemoptysis. Only 10% are asymptomatic at diagnosis [5]. Non-small cell lung cancer (NSCLC) is more common than small cell lung cancer. Adenocarcinoma is the most common subtype of NSCLC followed by squamous cell cancer. These histological classifications can be made by obtaining a tissue sample through multiple methods including sputum cytology, thoracentesis, excisional lymph node biopsy, transbronchial needle aspiration, transthoracic needle aspiration, video-assisted thoracoscopy, and thoracotomy. This case report evaluates a patient who presented to the emergency department (ED) with hemoptysis, who later developed a pneumothorax and subcutaneous emphysema following a lung biopsy.

## Case presentation

The patient was a 67-year-old male who presented initially with acute hemoptysis. He stated that he was coughing up mild-to-moderate amount of blood for two weeks and that it was worsening in amount and frequency. He also admitted to increased shortness of breath and a 14-pound weight loss over the last month. He denied chest pain, abdominal pain, headache, or dizziness. He had a medical history significant for oxygen-dependent chronic obstructive pulmonary disease (COPD). He denied any cardiac history, recent travel, or illness. His medications included budesonide/formoterol, tamsulosin, and ranitidine. He denied alcohol or illicit drug use. He had a history of tobacco use with a total of 50 pack-years of smoking, but he quit approximately 10 years ago. His father died at the age of 52 years from esophageal cancer.

**Figure 1:**
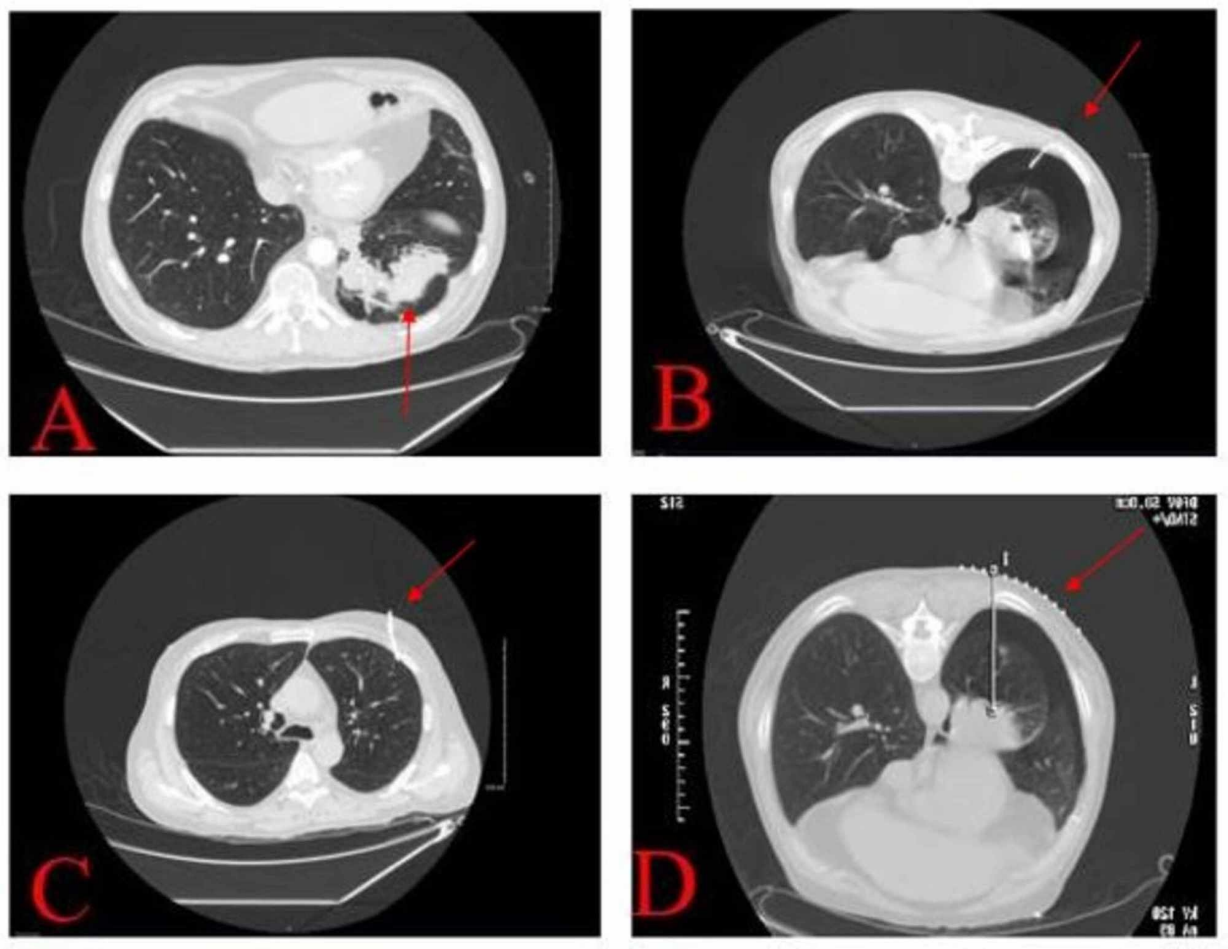
Series of CT scans of the patient (A) Initial contrast-enhanced, axial chest CT (lung window) illustrating a mass with irregular contour in the left lower lobe of the lung (arrow) and post-obstructive atelectasis. (B) Pneumothorax in the left lung during first attempted needle biopsy (arrow). (C) Trace pneumothorax after chest tube placed (arrow). Biopsy aborted. (D) Repeat biopsy successfully completed on day 2 after failed attempt and chest tube placement (arrow).

In the ED, his labs were significant for leukocytosis (11.02 K/mm3) and hemoglobin (11.6 gm/dL) without known baselines. He underwent a chest radiograph that showed hyperinflated lungs, small left pleural effusion, and pleural parenchymal scar/subsegmental atelectasis at the left lung base. This was followed by a CT angiogram of the chest, which was negative for pulmonary embolus but significant for a left lower lobe mass, post-obstructive atelectasis, and an infiltrate around the mass (Figure 1A). The mass measured as 5.6 cm x 5.7 cm. He did have several episodes of hemoptysis in the ED. He was admitted for further medical management of the new lung mass and ongoing hemoptysis.

**Figure 2:**
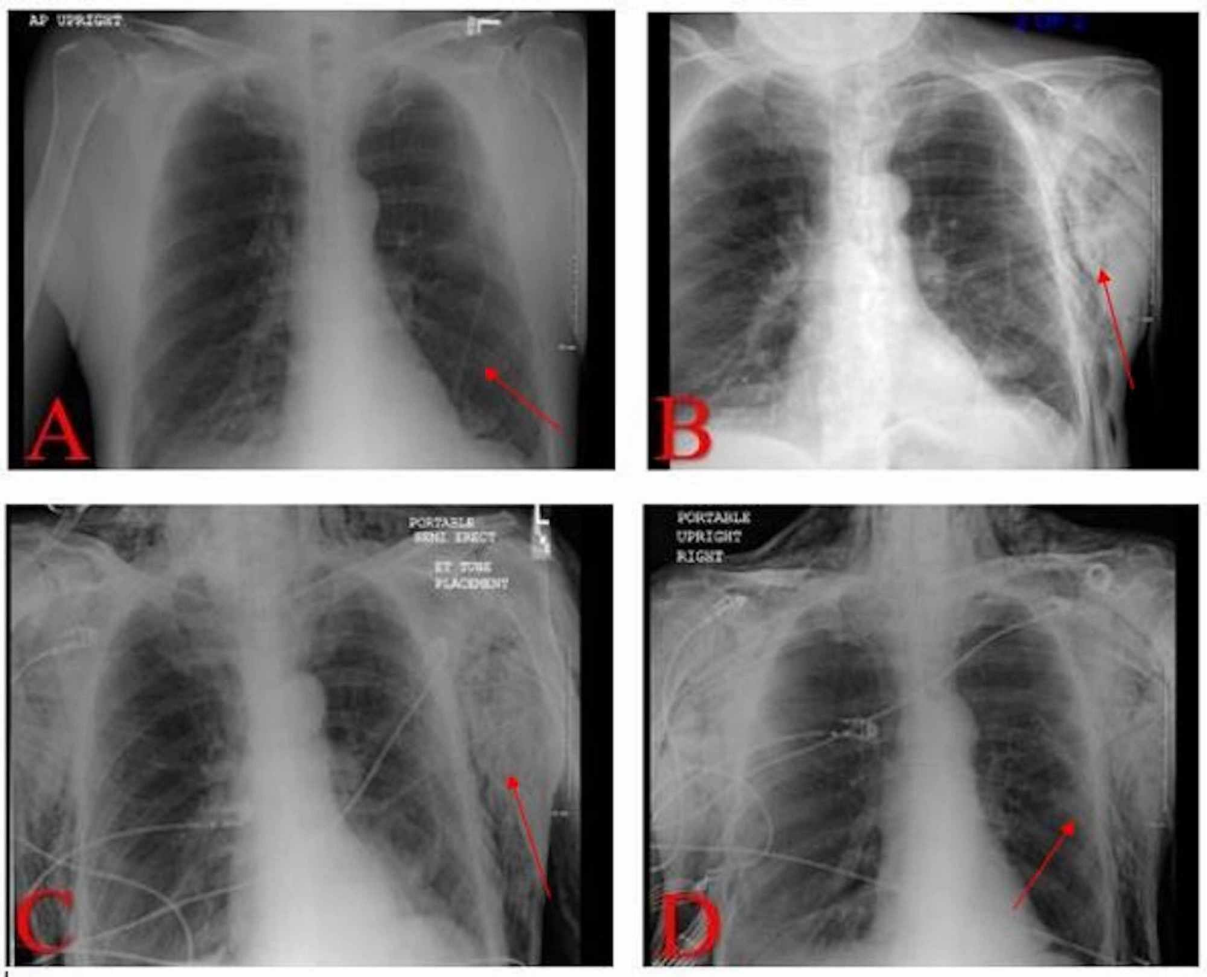
Series of chest X-rays of the patients (A) Chest X-ray on day 0 after failed biopsy. (B) Chest X-ray on day 1 after biopsy and day 3 after aborted biopsy: development of subcutaneous emphysema (arrow). (C) Chest X-ray on day 2 after biopsy and day 4 after failed biopsy, and rapid response was called and the patient was transferred to ICU for intubation: worsening subcutaneous emphysema (arrow), airway involvement. (D) Chest X-ray on day 2 after biopsy and day 4 after failed biopsy, with 24-French chest tube placement (arrow). ICU, intensive care unit

**Figure 3:**
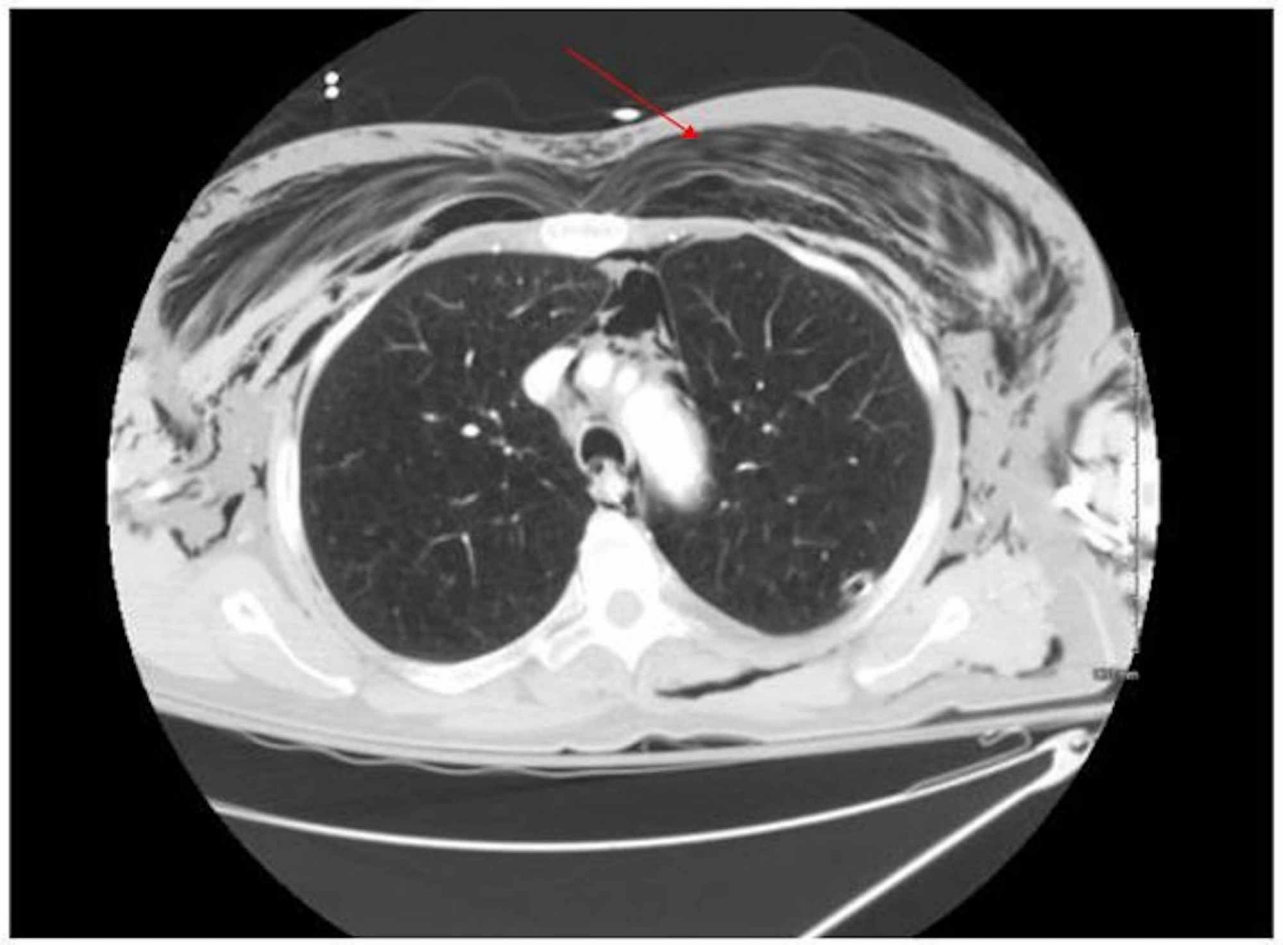
Repeat CT scan of the patient showing extensive subcutaneous emphysema Repeat CT on day 2 after biopsy and day 4 after failed biopsy, showing extensive subcutaneous emphysema (arrow) and pneumomediastinum, with the chest tube located in the posterior left lung.

During admission, a bronchoscopy was performed for the hemoptysis, which revealed significant narrowing of the left main bronchus. During suctioning, there was easy bleeding. The endobronchial brushing was significant for the presence of malignant cells, most likely squamous cell carcinoma; however, the final pathology was pending. A hematologist/oncologist was consulted, and a biopsy of the lung mass was arranged with tumor markers. The carcinoembryonic antigen (CEA) tumor marker came back elevated. During the attempted CT-guided needle biopsy, the patient developed a pneumothorax followed by respiratory distress (Figure 1B). The biopsy was aborted and the patient had a small-sized 12-French chest tube placed, which improved the pneumothorax and relieved the dyspnea (Figure 1C). Figure 2A shows the chest X-ray on the failed biopsy day. On the second day, another biopsy was performed, which was successful (Figure 1D). One day later, the patient developed massive subcutaneous air in the left lateral chest and cervical region (Figure 2B). Overnight, the patient developed a pressure sensation in his throat and reported having trouble swallowing. A rapid response was called, at which time he was moved to the medical intensive care unit for airway management and was intubated (Figure 2C). A larger 24-French mid axillary chest tube was placed, and the smaller first chest tube removed (Figure 2D). He underwent a repeat chest CT scan, which revealed extensive, bilateral subcutaneous emphysema and pneumomediastinum (Figure 3). Over the next several days, the patient’s subcutaneous emphysema gradually resolved, and a few days later, the chest tube was removed. He was discharged and referred to an oncologist for the management of his lung cancer.

## Discussion

Transthoracic needle biopsy through CT is commonly used as a diagnostic tool for lung cancer. The procedure has high diagnostic accuracy, sensitivity, and specificity. Collected pathological specimens are used for disease staging and driving appropriate treatment plans. However, this procedure is not without its risks, which include pulmonary hemorrhage and, most commonly, pneumothorax. Across the literature, iatrogenic pneumothorax secondary to CT-guided biopsy has an incidence rate of 17-30%, with roughly 14-29% of cases requiring intervention with a chest tube placement [6].

Certain patient populations and risk factors increase the propensity of iatrogenic pneumothorax. Increased age, history of COPD, and smoking are risk factors that have been associated with a higher incidence rate of pneumothorax. Other reported risk factors related to CT-guided biopsy include a long needle path to the lesion (>4 cm), lesion location (worse in parenchyma vs. lung pleura), needle angle, increased number of pleural punctures, supine position, and smaller lesion size [7].

Management of symptomatic iatrogenic pneumothorax is usually with the placement of an emergent chest tube. There has been a growing argument for the use of smaller caliber, defined as less than 14-French chest tubes, such as pigtail catheters, for the initial treatment of pneumothorax versus the more traditional, larger chest tubes. Multiple studies have illustrated no significant difference in success between larger and smaller chest tubes but did note a significant decrease in infection rate, overall complication rate, and pain at the site of insertion with the smaller chest tubes compared with large chest tubes [8].

Subcutaneous emphysema in the thoracic region is often a sequelae of an underlying thoracic condition. Penetrating or blunt trauma, infection by gas-forming organisms, and recent cardiothoracic surgery have all been described as causes for the development of subcutaneous emphysema which, at times, can serve as an alarming sign for a worsening underlying lung condition. It is important to note that perforation of other luminal structures, such as the bronchus, larynx, trachea, esophagus, and, very rarely, the colon, can also lead to a similar presentation of thoracic subcutaneous emphysema. Although often alarming in appearance, subcutaneous emphysema tends to follow a benign course with patients typically presenting with crepitus, dysphonia, and dysphagia. The subcutaneous gas may uniformly diffuse across the entirety of the thoracic region and can reach the upper extremities and cervicofacial regions, as presented in this case presentation. Although rare, increased pressure within the thoracic space can lead to serious complications, such as upper airway compression and vasculature compromise, requiring emergent intervention as necessary.

CT scans can clearly illustrate the presence and location of subcutaneous emphysema, often with air dissecting between the pectoral muscles, giving a streaking effect, as seen in the presented case, known as the ginkgo leaf sign [9]. This imaging finding, however, can obscure important underlying pathology such as a pneumothorax, an infiltrate, or other parenchymal disease. Because of the typical benign course of subcutaneous emphysema, patients are typically observed for any cardiopulmonary compromise and conservatively managed. If intervention is necessary, infraclavicular “blow hole” incisions and placement of fenestrated subcutaneous catheters have been described as safe and effective in evacuating air and relieving pressure [10].

## Conclusions

Our case reports unfortunate complications of a workup intended to help the diagnosis of lung cancer and direct the patient’s treatment. The new-onset hemoptysis in a former 50-pack-year smoker is highly concerning for malignancy, which was also suspected based on imaging. The risk factors and lung mass location increased the risk of complication during the needed transthoracic needle biopsy. This case report highlights the critical thinking required for the workup of new hemoptysis and the subsequent management of the complications experienced. A large-sized chest tube may be preferred in such circumstances.
